# Organ‐contour‐driven auto‐matching algorithm in image‐guided radiotherapy

**DOI:** 10.1002/acm2.14220

**Published:** 2023-11-23

**Authors:** Yukako Kishigami, Mitsuhiro Nakamura, Hiroyuki Okamoto, Ayaka Takahashi, Hiraku Iramina, Makoto Sasaki, Kohei Kawata, Hiroshi Igaki

**Affiliations:** ^1^ Department of Advanced Medical Physics Graduate School of Medicine Kyoto University Kyoto Japan; ^2^ Radiation Safety and Quality Assurance Division National Cancer Center Hospital Tokyo Japan; ^3^ Department of Radiation Oncology National Cancer Center Hospital Tokyo Japan; ^4^ Department of Radiation Oncology and Image‐Applied Therapy Kyoto University Kyoto Japan; ^5^ Division of Clinical Radiology Service Kyoto University Hospital Kyoto Japan

**Keywords:** inter‐observer variability, organ‐contour‐driven auto‐matching, soft‐tissue

## Abstract

**Purpose:**

This study aimed to demonstrate the potential clinical applicability of an organ‐contour‐driven auto‐matching algorithm in image‐guided radiotherapy.

**Methods:**

This study included eleven consecutive patients with cervical cancer who underwent radiotherapy in 23 or 25 fractions. Daily and reference magnetic resonance images were converted into mesh models. A weight‐based algorithm was implemented to optimize the distance between the mesh model vertices and surface of the reference model during the positioning process. Within the cost function, weight parameters were employed to prioritize specific organs for positioning. In this study, three scenarios with different weight parameters were prepared. The optimal translation and rotation values for the cervix and uterus were determined based on the calculated translations alone or in combination with rotations, with a rotation limit of ±3°. Subsequently, the coverage probabilities of the following two planning target volumes (PTV), an isotropic 5 mm and anisotropic margins derived from a previous study, were evaluated.

**Results:**

The percentage of translations exceeding 10 mm varied from 9% to 18% depending on the scenario. For small PTV sizes, more than 80% of all fractions had a coverage of 80% or higher. In contrast, for large PTV sizes, more than 90% of all fractions had a coverage of 95% or higher. The difference between the median coverage with translational positioning alone and that with both translational and rotational positioning was 1% or less.

**Conclusion:**

This algorithm facilitates quantitative positioning by utilizing a cost function that prioritizes organs for positioning. Consequently, consistent displacement values were algorithmically generated. This study also revealed that the impact of rotational corrections, limited to ±3°, on PTV coverage was minimal.

## 1 INTRODUCTION

Image‐guided radiotherapy (IGRT) has become indispensable in the field of external beam radiotherapy owing to its effectiveness in compensating for patient positioning errors.^1^, ^2^ Among various image guidance functionalities, cone beam computed tomography (CBCT) and magnetic resonance (MR) imaging are the mainstream image guidance functionalities in IGRT, enabling soft‐tissue matching.^3^, ^4^, ^5^ Several investigators have demonstrated that daily soft‐tissue matching resulted in smaller planning target volume (PTV) margins, outperforming bone‐ or skin‐based matching methods.^6^, ^7^ These findings highlight the effectiveness of soft‐tissue matching as a valuable approach for compensating for daily variations in target position.

However, soft‐tissue matching faces several difficulties. Previous studies have consistently identified inter‐ and intra‐observer variability as great issues in soft‐tissue matching.^3^, ^8^, ^9^ Hirose et al. emphasized the importance of considering inter‐observer variability when determining the clinical target volume for PTV margins.^3^ Sasaki et al. demonstrated substantial inter‐observer variability and increased time requirements for soft‐tissue matching in pancreatic cancer, particularly among trainees with limited experience in IGRT.^8^ Furthermore, Zhang et al. conducted an analysis of the impact of inter‐observer variability, underscoring its greater influence on organs at risk (OARs) than on the target in prostate cancer.^9^

Current radiotherapy systems are unable to overcome the well‐acknowledged challenges associated with soft‐tissue matching. To address these issues, automation must be implemented. In this study, we focused on utilizing organ contours to achieve automation. Several auto‐segmentation techniques have been introduced in the field of radiotherapy to assist in the delineation of targets and OARs.^10^, ^11^ Furthermore, deep learning‐based auto‐segmentation has demonstrated its utility in planning image and daily image segmentation.^12^ While the current segmentation accuracy is imperfect, ongoing technological advancements are expected to result in improved accuracy and the eventual achievement of highly precise auto‐segmentation capabilities for daily images.^11^, ^13^

The primary objective of this study is to demonstrate the potential clinical applicability of an organ‐contour‐driven auto‐matching algorithm in IGRT, laying the groundwork for a future in which daily contouring will become a practical reality. The implementation of auto‐matching technology is expected to enhance throughput and revitalize the field of radiotherapy.

## 2 MATERIALS AND METHODS

### 2.1 Patients and data preparation

For the algorithm development, eleven consecutive patients with cervical cancer who underwent MR‐guided intensity‐modulated radiotherapy using the ViewRay MRIdian system (ViewRay Inc., Oakwood, OH, USA) were included. The treatment was administered in 23 or 25 fractions. Detailed patient information is presented in Table 1. In this study, volumetric imaging was performed using true‐fast imaging with a steady‐state precession sequence. A total of 273 datasets were analyzed. The acquired MR scans had a slice thickness of 3 mm and pixel dimensions of 1.5 mm × 1.5 mm. Daily MR images were co‐registered with the planned MR images using the pelvic bones as a reference to determine the original position of the cervix and uterus. A single radiation oncologist manually delineated the contours of the cervix and uterus on the daily MR images. These contours were converted into the mesh file format using a commercially available system (ITEM Viewer Planning and Assistant System, ITEM Corporation, Osaka, Japan). Subsequently, the reference and daily mesh models represented by the vertices and triangular meshes were generated. The study protocol was approved by the institutional review board (approval number: 2020‐556).

**TABLE 1:** Patient characteristics.

Pt#	Age (y.o.)	Pathology	TNM	Stage	Chemo	Dose (Gy/fr)	Cx (cm^3^)^a^	Ut (cm^3^)^a^
1	63	Ad	T3bN1M0	IIIB	–	46/25	15.9	102.1
2	35	SCC	T3bN1M0	IIIB	CDDP	45/25	103.8	77.9
3	65	SCC	T1b1N0M0	IB	–	45/25	9.9	3.6
4	73	SCC	T3bN1M0	IIIB	–	46/23	15.5	27.2
5	76	SCC	T3bN1M0	IIIB	CDDP	45/25	103.5	78.9
6	67	SCC	T2bN1M0	IIB	CDDP	45/25	38.8	16.9
7	71	SCC	T2bN1M0	IIB	CBDCA	45/25	29.6	33.4
8	70	SCC	T2bN0M0	IIB	CDDP	45/25	20.3	13.6
9	47	SCC	T3bN1M0	IIIB	CDDP	45/25	46.9	82.9
10	88	SCC	T1b1N0M0	IB	–	45/25	13.7	21.7
11	44	SCC	T3bNxM0	IIIB	CBDCA	45/25	45.0	91.2

Abbreviations: Ad, adenocarcinoma; CBDCA, carboplatin; CDDP, cisplatin; chemo, chemotherapy; Cx, cervix; Pt#, patients; SCC, squamous cell carcinoma; Ut, uterus.

* ^a^Volume at treatment planning (cm^3^).

### 2.2 Cost function

In this study, the vertices of the mesh models generated from organ contours were used for auto‐matching. For each treatment fraction, the vertices of the daily cervical and uterine mesh models were categorized into two groups: vertices located outside the reference model (referred to as “outside vertices”) and vertices situated within the reference model (referred to as “inside vertices”). This study involved obtaining the distances between the outside or inside vertices and the surface of the reference model. These distances were denoted as “*d*” for the outside vertices and “*D*” for the inside vertices. The cost of organ A was defined as $\;\mathrm{Cos}t_{A}=\;W_{\mathrm{out}}\sum d_{i}^{2}/i+W_{\mathrm{in}}\sum D_{t}^{2}/t$. The number of outside and inside vertices were denoted as *i* and *t*, respectively. A weight *W*_out_ was applied to the outside vertices, encouraging them to move closer to the reference position and align with the reference model. Similarly, a weight *W*_in_ was applied to the inside vertices to promote their alignment with the reference position. The cost function used in this study was the sum of the costs associated with the cervix and uterus. In this study, three scenarios with different weight parameters were prepared.

#### 2.2.1 Scenario A

All weights, *W*_out_ and *W*_in_ for the cervix (*W*_out_cervix_ and *W*_in_cervix_) and *W*_out_ and *W*_in_ for the uterus (*W*_out_uterus_ and *W*_in_uterus_), were set to one.

#### 2.2.2 Scenario B

A higher weight was assigned to the outside vertices of the cervix and uterus to bring them closer to the reference position. The weights were set as follows: (*W*_out_cervix_, *W*_in_cervix_, *W*_out_uterus_, *W*_in_uterus_) = (10, 5, 10, 5).

#### 2.2.3 Scenario C

The highest weight was assigned to *W*_out_cervix_, and the weights were prioritized in the following order: (*W*_out_cervix_, *W*_in_cervix_, *W*_out_uterus_, *W*_in_uterus_) = (10, 5, 2, 0.5). The reason why different weights were used for the cervix and uterus was to focus on the alignment of the cervix to the reference position.^14^

Scenario A represented the condition in which no additional weight was employed. This scenario served as the reference to assess whether the inclusion of weight improved positioning or not. When selecting the weights, it was crucial to consider the relative size of the weight sets as the specific weight values themselves were less significant.

### 2.3 Optimization of cost function

**FIGURE 1:**
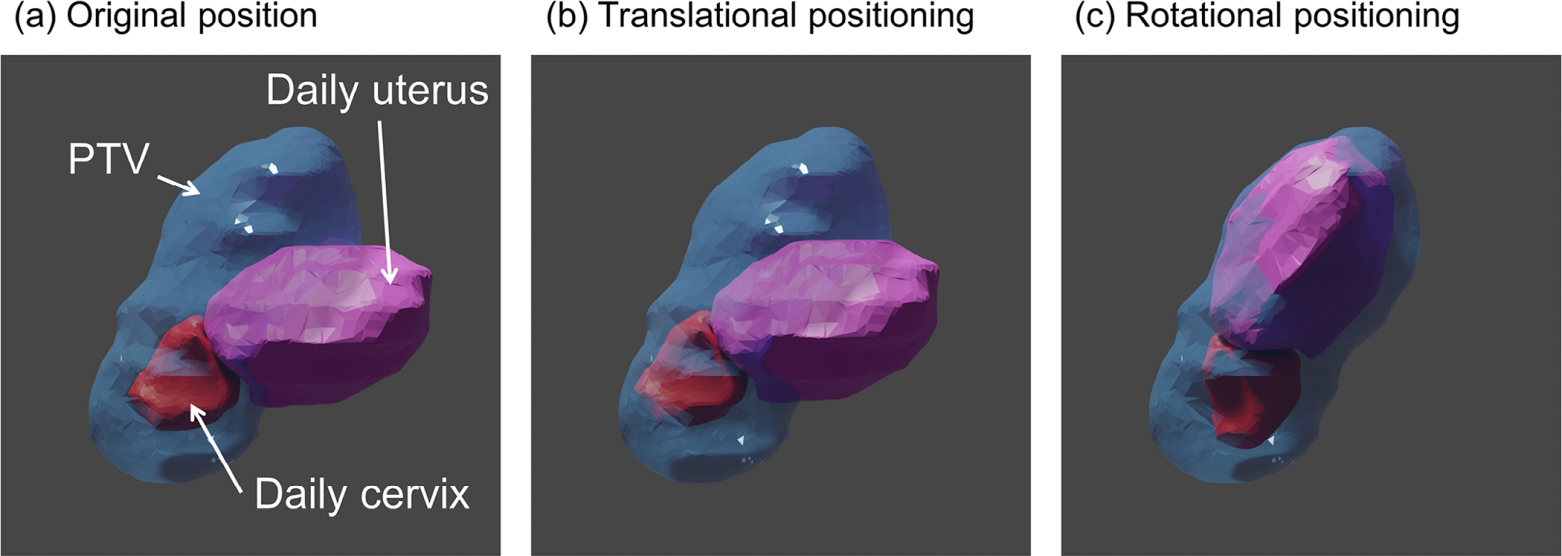
Original position (a), the position after translational positioning (b), and the position after rotational positioning (c). The daily cervix, daily uterus, and PTV are represented by the red, pink, and blue structures, respectively.

In this study, the concept of dichotomy was used to determine the optimal translation and rotation values. Before the initial optimization, the original cost was calculated based on the original position determined through co‐registration, using the pelvic bones as a reference. During each optimization, the calculation was performed for translational positioning only. Once translational positioning was completed, the calculation was then performed for rotational positioning, focusing solely on the rotations (Figure 1).

#### 2.3.1 Translational positioning

- At the first optimization of translation, the entire cervix and uterus were shifted by the distance between the centroid of the daily mesh model and reference model.
- The *M*th translational value (*M*th translation; *M* ≥ 2) was defined as half the distance of the (*M*‐1)th translation. If *M*th translation was less than 1 mm, which represents the minimum requirement of the translational accuracy of the couch,^15^ *M*th and subsequent translation was set to 1 mm.
- Following each translation, the cost was calculated and compared with that before translation. If the cost did not decrease, the cervix and uterus were returned to the position before translation.
- If the cost did not decrease after translation of 1 mm, the translational positioning process was completed. At this point, the total amount of translation from the original position was calculated. Subsequently, the rotational positioning phase commenced.

#### 2.3.2 Rotational positioning

- At the first optimization of rotation, the entire cervix and uterus were rotated by 45°.
- The *M*th rotational value (*M* ≥ 2) was defined as half the rotational angle from the (*M*‐1)th rotation. If *M*th rotational angle was less than 0.5°, which represents the minimum requirement of the rotational accuracy of the couch,^15^ *M*th and subsequent rotational angle was set to 0.5°.
- Following each rotation, the cost was calculated and compared with that before rotation. If the cost did not decrease, the cervix and uterus were returned to the position before rotation.
- If the cost did not decrease after rotation of 0.5°, the rotation positioning process was completed. At this point, the total rotation from the position where translational positioning was completed was then calculated.

**FIGURE 2:**
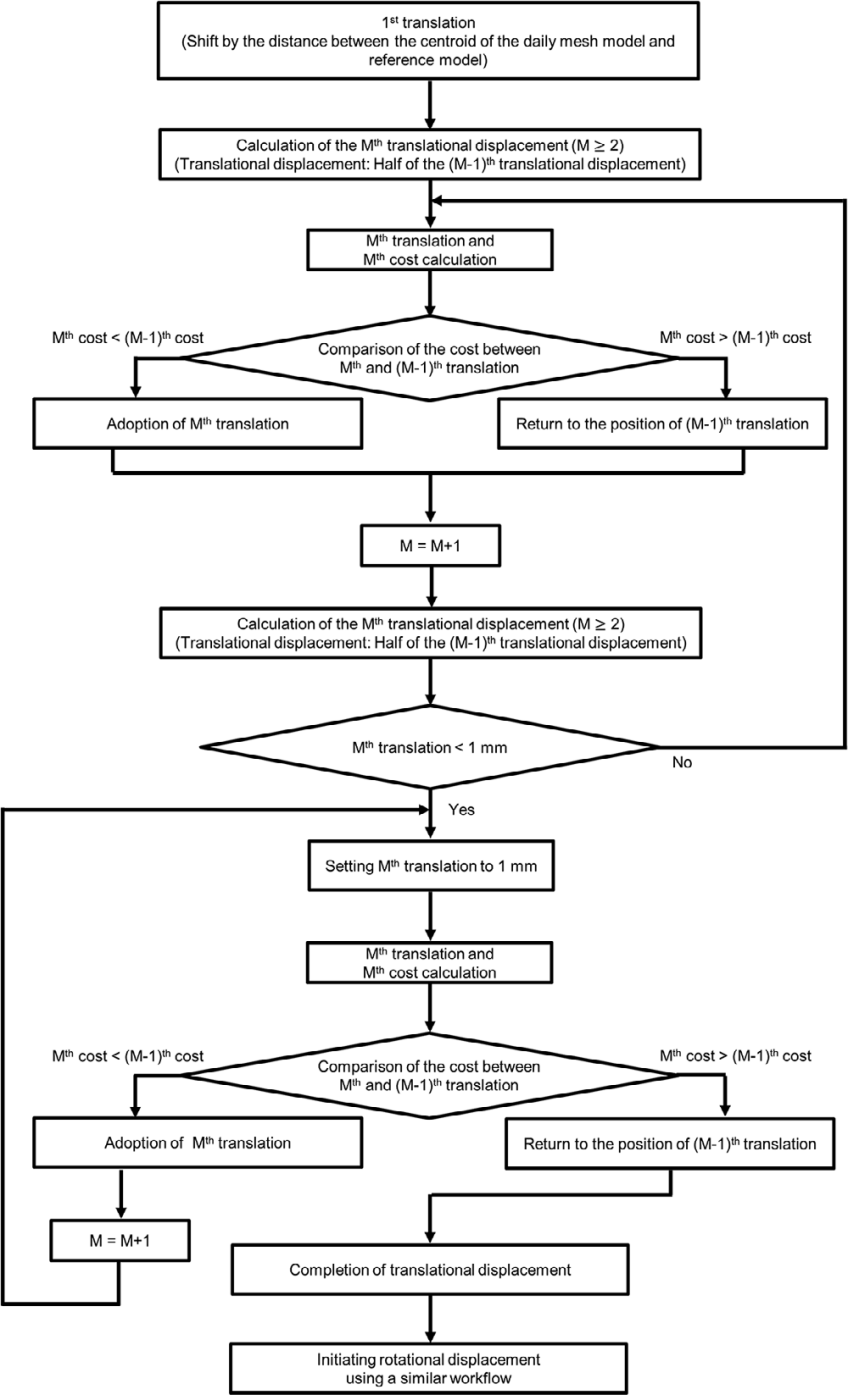
Flow of optimization.

The flow of optimization was illustrated in Figure 2.

### 2.4 Calculation of PTV coverage after optimization

In addition to the primary focus on developing the algorithm, we secondarily explored its impact on PTV coverage. Following each optimization for the three weight sets, the positions of the cervix and uterus were adjusted based on the displacements calculated from either translational positioning alone or both translational and rotational positioning. Subsequently, the coverage probabilities of the PTV were assessed for all eleven patients. For rotational positioning, the rotation limit was set to 3°, which corresponds to the allowable couch rotation tolerance at our institution. The coverage was assessed by determining the ratio of vertices from the daily cervical and uterine shape models located within the PTV to the total number of vertices outside the PTV. The PTV margins used in this study were as follows: (1) an isotropic 5 mm (PTV_iso_) and (2) anisotropic (right (R), left (L), anterior (A), posterior (P), superior (S), inferior (I)) margins of (5, 5, 15, 15, 10, 10) mm for the cervix and (10, 10, 20, 20, 15, 15) mm for the uterus (PTV_aniso_). The anisotropic margins of the uterus were derived from a previous study.^16^ For the cervix, the margins were defined as 5 mm smaller than the uterine margins.

### 2.5 Statistical analysis

To evaluate the statistical differences in translation and rotation among scenarios *A*, *B*, and *C*, a paired *t*‐test was utilized. Bonferroni correction was applied for multiple comparisons. Additionally, the PTV coverage with both translational positioning alone and combined translational and rotational positioning was also analyzed using *t*‐tests. The significance level was set at 0.05. The calculation was done by Microsoft Excel 2019.

### 2.6 Experiment environment

All processes in this study were carried out on a desktop computer equipped with a graphics processing unit (CPU, Intel (R) with 3.00 GHz; RAM, 256 GB; GPU, NVIDIA RTX A5000) running CUDA 11.4 and Python 3.8.10.

## 3 RESULTS

### 3.1 The amount of translational and rotational corrections

**FIGURE 3:**
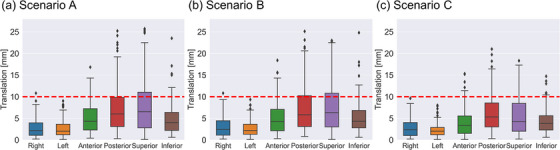
Translations for scenario A (a), B (b), and C (c) calculated by the optimization for all fractions. The red dashed line represents a translation of 10 mm.

The percentage of translations exceeding 10 mm varied depending on the scenario (Figure 3). The posterior and superior directions exhibited higher frequencies of these occurrences. In scenarios A and B, translations exceeding 10 mm were observed in 14% of all fractions in the posterior direction, whereas in the superior direction, the corresponding percentages were 18%. In scenario C, translations exceeding 10 mm accounted for 11 and 9% of all fractions in the posterior and superior directions, respectively. In contrast, translations exceeding 10 mm were only required in 0.4% of all fractions (one out of 273 fractions) for the right and left directions in scenarios A and B. Conversely, no fractions in scenario C exhibited translations exceeding 10 mm. Significant differences were observed in the anterior between scenarios A and C and superior directions between scenarios A and C and between scenarios B and C (*p* < 0.05), although larger translational corrections were required in scenarios A and B than in scenario C. However, no significant differences were observed in translations in any of the six directions between scenarios A and B.

**FIGURE 4:**
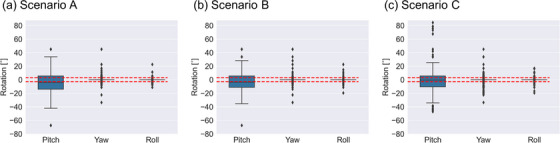
Rotations for scenario A (a), B (b), and C (c) calculated by the optimization for all fractions. The red dashed lines represent a rotation of ±3°.

In terms of rotation, a consistent trend was observed across all scenarios (Figure 4). The largest rotation was observed in the pitch axis, with median values (interquartile range) of −3.8 (−14.0−5.6), −3.8 (−11.2−5.6), and −0.7 (−10.8−5.5) for scenarios A, B, and C, respectively. Conversely, the median and interquartile ranges for the yaw and roll axes were zero in all scenarios.

### 3.2 PTV coverage

**FIGURE 5:**
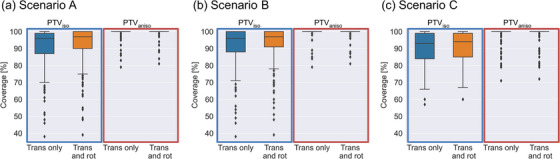
Results of PTV coverage for scenario A (a), B (b), and C (c) are presented. The coverage of PTV_iso_ and PTV_aniso_ is indicated by a blue and red rectangle, respectively. Within each rectangle, the left side represents the coverage of translational positioning only (denoted as “Trans only”), and the right side represents that of both translational and rotational positioning (denoted as “Trans and rot”).

Figure 5 shows the results of PTV coverage for scenarios A, B, and C. The PTV_iso_ had a coverage of 80% or higher for more than 80% of all fractions. In contrast, PTV_aniso_ had a coverage of 95% or higher for more than 90% of all fractions. While significant differences were observed (*p* < 0.05), the difference between the median coverage with translational positioning alone and that with both translational and rotational positioning was 1% or less.

Among scenarios A, B, and C, scenarios A and B exhibited higher coverage for both PTV_iso_ and PTV_aniso_ than that of scenario C. However, a few outliers with lower coverage were observed for PTV_iso_. In all scenarios, 8%−16% of all fractions exhibited less than 80% coverage for PTV_iso_, regardless of rotational correction. Conversely, 1% or less of all fractions had less than 80% coverage for PTV_aniso_.

## 4 DISCUSSION

### 4.1 Inter‐observer variability

As shown in supplementary materials, regarding translation, the standard deviation exceeded 5 mm for 4% of the entire dataset, regardless of the direction. With respect to rotation, the standard deviation exceeded 3° for 6% of the entire dataset, regardless of the axis. This study also observed inter‐observer variability, consistent with findings from previous studies.^3^, ^8^, ^9^, ^17^, ^18^, ^19^ These findings emphasize the need for auto‐matching techniques to minimize inter‐observer variability and achieve consistent soft‐tissue matching.

### 4.2 Validity of the calculated translations by the algorithm

Although co‐registration based on the pelvic bones was performed, translations exceeding 10 mm were still observed in specific directions across all weight sets. This highlights the inherent variability of the cervix and uterus, as discussed in previous studies.^16^, ^20^, ^21^, ^22^ Translations exceeding 10 mm were more frequently observed in the posterior and superior directions, whereas smaller translations were observed in the right and left directions, consistent with previous findings.^23^, ^24^ Thus, the translations calculated using the algorithm were considered valid.

### 4.3 Clinical significance of the displacement calculated by the algorithm

First, we discuss the findings regarding the correction of the translational component. Among the three scenarios used, scenarios A and B placed greater emphasis on correcting the position of the uterus than scenario C. As a result, adjustments were more evident in the anterior and superior directions. This observation can be attributed to the fact that the uterus exhibits greater mobility than the cervix.^24^

Next, regarding the correction of the rotational components, a pitch of 3° or higher was necessary in several fractions (Figure 4). However, our hospital imposes a limit of 3° for couch rotation for safety reasons. Consequently, adequate rotational corrections may be unattainable in certain clinical scenarios. Conversely, minimal rotational movement of the cervix and uterus was observed along the yaw and roll axes, with a median value of 0. This highlights the importance of incorporating anisotropic margins to compensate for the pitch rotation. Scenario C, which prioritized the cervix over the uterus, yielded the lowest median pitch value. This indicates that addressing uncertainties related to the uterus requires substantial attention to correct pitch rotations.

### 4.4 Impact of the algorithm on PTV coverage

**FIGURE 6:**
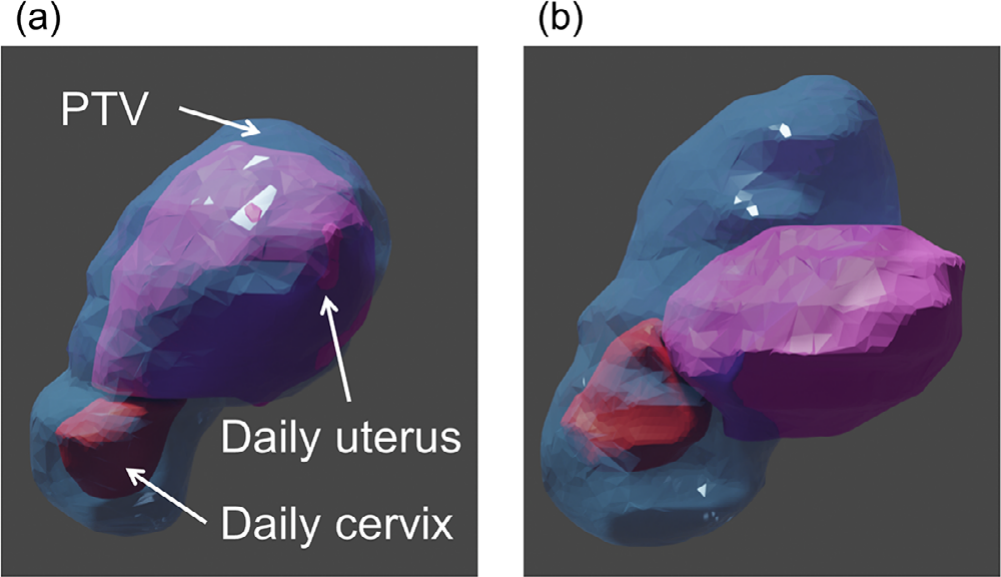
Comparison of the uterus position after translational positioning for a case with similar shapes (a) and with large different shapes (b), compared to the reference shapes. The daily cervix, daily uterus, and PTV are represented by the red, pink, and blue structures, respectively.

In this study, the impact of the developed algorithm was secondarily evaluated in terms of PTV coverage. In scenarios A and B, we aimed to position the cervix and uterus equally within the PTV. However, some outliers were observed (Figure 5), indicating that the uterus had different shapes compared to the reference position and was largely located outside the PTV (Figure 6). Priority was given to align the uterus closer to the reference position, but as a result, the cervix position deviated from the reference position. Thus, when the uterus has a different shape or position than the reference, it may be necessary to prioritize the alignment of a target with higher malignancy, the cervix rather than the uterus (in this context), or further expand the margin.

The impact of rotational corrections limited to ±3° on PTV coverage was minimal for PTV_aniso_ (Figure 5). As mentioned previously, rotational corrections of 3° or more were deemed critical in certain fractions, implying that corrections below 3° may have a negligible effect. Furthermore, PTV_iso_ exhibited instances of insufficient coverage, even after correcting for rotational components. Therefore, precise margin settings are crucial to compensate for the rotational components of cervical cancer.

### 4.5 Importance of organ‐driven auto‐matching

Our approach algorithmically yields consistent displacement values. The integration of auto‐matching technology is pivotal for mitigating inter‐observer variability. Additionally, it offers the potential to minimize the risk of irradiating incorrect areas due to misidentification. This aspect becomes particularly critical in cases such as spinal stereotactic body radiation therapy, where structures resembling the target can complicate the visual identification of the precise irradiation area. These challenges increase the possibility of erroneous irradiation of incorrect targets. The utilization of the organ‐contour‐driven auto‐matching algorithm presents a valuable solution for mitigating the risk of mis‐irradiation caused by misrecognition. This advanced approach elevates the safety standards of radiotherapy and contributes to its progress. By diminishing inter‐observer variability and enhancing the precision of target identification, auto‐matching technology ensures treatments that are more accurate and dependable. Ultimately, this brings benefits to both patients and healthcare professionals, fostering improved radiotherapy outcomes.

### 4.6 Limitations

In this study, there were four limitations that warranted careful consideration. Firstly, the algorithm relies on organ contours for its function. Although auto‐contouring technology has made great progress in recent years and has become feasible,^11^, ^13^ its accuracy is imperfect. However, as mentioned above, it is anticipated that this challenge will be overcome in the near future and that the algorithm can be applied in conjunction with advanced contouring technology to reduce inter‐observer variability and minimize mis‐irradiation. Furthermore, the emergence of real‐time auto‐segmentation offers promising prospects for further enhancing the efficacy of this algorithm. The integration of real‐time auto‐segmentation has the potential to improve the overall performance and reliability of the algorithm, contributing to more precise and accurate patient positioning during radiotherapy. Secondly, the absence of ground truth makes it uncertain whether the algorithm is producing accurate results. Nevertheless, this mirrors the situation encountered in clinical practice. Although some outliers were observed (Figure 5), it was determined that they resulted from considerable changes in the position of the uterus. However, in all other cases, visual evaluation confirmed that the daily cervix and uterus are consistently more centrally located within the PTV, indicating no major issues. Third, the weight sets presented in this study were arbitrarily chosen, and it is uncertain whether they are suitable for matching with other diseases. When applied clinically, it is preferable for users to determine weight sets based on their facility's background and treatment policies, considering the specific disease. Finally, the algorithm‐based soft‐tissue matching calculation typically consumed around 10 min. Two primary factors significantly influenced the calculation time. One was the number of vertices in the mesh model for which, we did not set an upper limit in this study. Therefore, implementing an upper limit on the number of vertices would enhance efficiency and reduce calculation time. The other factor was the computational environment. However, with advancements in computer technology, the calculation time is expected to decrease significantly.

## 5 CONCLUSION

We proposed the organ‐contour‐driven auto‐matching algorithm and demonstrated its potential clinical applicability in IGRT. This algorithm facilitates quantitative positioning by utilizing a cost function that prioritizes organs for positioning. Consequently, consistent displacement values were algorithmically generated. Additionally, we also found that the impact of rotational corrections, limited to ±3°, on PTV coverage was minimal when the PTV margins were large enough to compensate for the variability of cervix and uterus.

## AUTHOR CONTRIBUTIONS

Y.K. and M.N. planned the study. Y.K. performed the statistical analysis and drafted the manuscript. H.O., A.T., H.I., S.M., K.K., and H.I. helped draft the manuscript. All authors read and approved the final manuscript.

## CONFLICT OF INTEREST STATEMENT

We have no financial relationships to disclose.

## Supporting information

Supporting Information

## Data Availability

The data that support the findings of this study are not available.
