# Supplementary material for: Organ‐contour‐driven auto‐matching algorithm in image‐guided radiotherapy
Source: J Appl Clin Med Phys. 2023 Nov 23;25(1):e14220. doi: 10.1002/acm2.14220 (PMC10795436; doi:10.1002/acm2.14220)
Supplement: Supplementary file 1 — Supporting Information [file ACM2-25-e14220-s001.docx]

**Supplementary materials**

**Materials and methods**

***Assessment of inter-observer variability***

As previously mentioned, inter-observer variability was identified in previous studies [1–3]. In this preliminary study, we further investigated inter-observer variability that might arise when performing soft-tissue matching with our facility's personnel and equipment. The soft tissue matching was conducted by three medical physicists and two radiation therapists. The three physicists had different levels of experience with 18, 8, and 3 years of experience, respectively. Similarly, the two radiation therapists had varying levels of experience with 13 and 3 years of experience, respectively. Soft-tissue matching was performed on six patients with pancreatic cancer, totaling 29 fractions. Planning CT (pCT) scans were acquired under breath-holding conditions using a 64-slice CT scanner (SOMATOM Definition AS; Siemens Healthineers, Erlangen, Germany). The CT scans were acquired at 120 kV, with a slice thickness and slice interval of 2.0 mm and field of view of 500 mm. Daily CBCT images were acquired using a Varian Ethos system (Varian Medical Systems, Palo Alto, CA, USA). The CBCT scans were performed at 125 kV with a slice thickness of 2.0 mm and scan diameter of 492 mm. The pCT and daily CBCT images were aligned based on the bony structures, and the participants were instructed to align the gross tumor volume (GTV) by performing translational and rotational adjustments of the daily CBCT images on the pCT source images. No movement limitations were imposed, and only the contour of the GTV delineated on the pCT was displayed. Inter-observer variability was defined as the standard deviation of each subject's positioning, calculated for each fraction of translation and rotation.

**Results**

***Assessment of inter-observer variability***

**Supplementary Figure 1** illustrates the results of inter-observer variability for translation and rotation. For translation, the standard deviation exceeded 2, 3, and 5 mm for 13, 5, and 4% of the entire dataset, respectively, regardless of the direction. For rotation, the standard deviation exceeded 2 and 3° for 13 and 6% of the entire dataset, respectively, regardless of the axis.

**References**

1. Hirose T, Arimura H, Fukunaga J, et al. Observer uncertainties of soft tissue-based patient positioning in IGRT. *J Appl Clin Med Phys* 2020;21:79-81. doi: 10.1002/acm2.12817.
2. Sasaki M, Nakamura M, Ashida R, et al. Assessing target localization accuracy across different soft-tissue matching protocols using end-exhalation breath-hold cone-beam computed tomography in patients with pancreatic cancer. *J Radiat Res* 2023;048. doi: 10.1093/jrr/rrad048.
3. Zhang X, Wang X, Li X, et al. Evaluating the impact of possible interobserver variability in CBCT-based soft-tissue matching using TCP/NTCP models for prostate cancer radiotherapy. *Radiat Oncol* 2022;17:62. doi: 10.1186/s13014-022-02034-1.

**
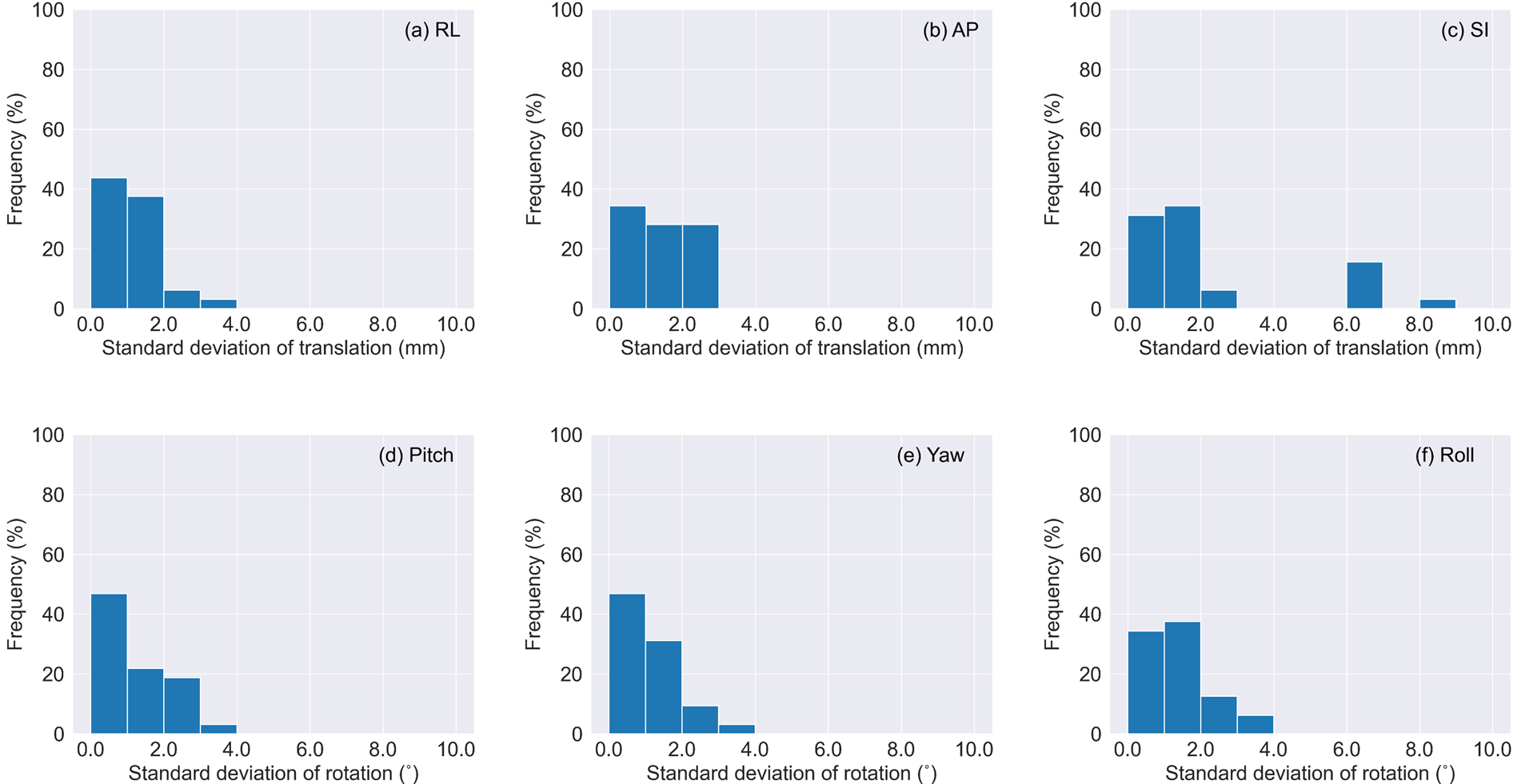
**

**Supplementary Figure 1.** The standard deviation results for translation ((a)-(c)) and rotation ((d)-(f)) representing inter-observer variability.
